# 伴JAK3基因突变的普通变异型免疫缺陷病1例

**DOI:** 10.3760/cma.j.issn.0253-2727.2022.01.017

**Published:** 2022-01

**Authors:** 薇 蔡, 芳婷 王, 亮亮 马

**Affiliations:** 大连医科大学附属第一医院血液科，大连 116011 Hematology Department, the First Affiliated Hospital of Dalian Medical University, Dalian 116011, China

患者，男，20岁，因“反复瘀斑3个月”于2020年10月就诊于我院。患者于入院前3个月无明显诱因出现皮肤多处瘀斑，可自行消退，未在意。此后反复发作，2个月前就诊于外院，血常规：WBC 8.41×10^9^/L，HGB 162 g/L，PLT 5×10^9^/L，免疫球蛋白IgG 4.57 g/L（正常参考值7～16 g/L），IgA 0.38 g/L（正常参考值0.7～4.0 g/L），IgM 0.36 g/L（正常参考值0.4～2.3 g/L），完善骨髓穿刺等检查诊断“免疫性血小板减少症”，给予重组人促血小板生成素（TPO）15 000 U/d 联合艾曲泊帕25 mg/d口服，2周后血小板计数升至160×10^9^/L，此后以艾曲泊帕25 mg/d维持治疗。2020年10月瘀斑再发而入住我院。起病以来无发热，否认肝病史和特殊家族史。入院后复查血常规：WBC7.05×10^9^/L，HGB 157 g/L，PLT 6×10^9^/L。网织血小板31％，球蛋白20.2 g/L，IgG 5.15 g/L，IgA 0.54 g/L，IgM 0.38 g/L，肝炎病毒学、甲状腺功能、风湿免疫相关抗体、淋巴细胞亚群、调节性T细胞（Treg）比例均无异常。流式细胞术检测血浆T细胞相关细胞因子（IL-2、IL-4、IL-6、IL-10、TNF-α、INF-γ和IL-17A）浓度无异常。肺CT和腹部彩超无异常。骨髓细胞形态学检查：增生活跃，粒系、红系、淋巴系形态和比例未见异常，全片见巨核细胞1515个，颗粒巨核细胞为主，未见产板巨核细胞，提示巨核细胞成熟障碍。染色体核型46,XY[4]。追问病史，患者自幼体弱，10岁前几乎每月均发生“感冒或发热”，患者近3年多次血生化检查示球蛋白14～18 g/L（正常参考值20～40 g/L）。患者父母均无免疫球蛋白和血常规异常。

采集患者及其父母静脉血样本，应用二代测序（NGS）技术筛查近700个血液和免疫系统遗传性疾病基因，发现患者JAK3基因存在一处杂合突变，遗传自其父亲，Sanger测序证实为19号染色体11号外显子发生错义突变c.1503 G>T（图1），该突变导致JH3结构域的p.Q501H氨基酸发生改变。最终诊断：普通变异型免疫缺陷病伴继发性免疫性血小板减少症。予静脉免疫球蛋白0.4 g·kg^−1^·d^−1^治疗并继续艾曲泊帕口服，2 d后血小板计数升至180×10^9^/L。遂停用静脉免疫球蛋白，继续艾曲泊帕口服维持治疗。2周后，血小板计数再次降至10×10^9^/L，加用TPO疗效不佳，改以阿伐曲泊帕20 mg/d联合环孢素A 100 mg/d、泼尼松10 mg/d口服治疗，2周后血小板计数恢复正常。此后多次复查，血小板计数为（56～100）×10^9^/L。

**图1:**
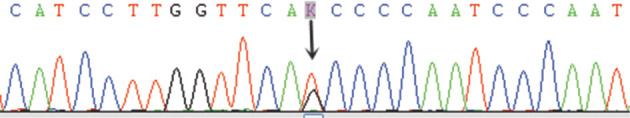
普通变异型免疫缺陷病患儿JAK3基因外显子11错义突变c.1503 G>T

